# Digital Divide in Online Education During the COVID-19 Pandemic: A Cosmetic Course From the View of the Regional Socioeconomic Distribution

**DOI:** 10.3389/fpubh.2021.796210

**Published:** 2022-01-03

**Authors:** Mengmeng Sun, Lidan Xiong, Li Li, Yu Chen, Jie Tang, Wei Hua, Yujie Mao

**Affiliations:** ^1^School of Medicine, University of Electronic Science and Technology of China, Chengdu, China; ^2^Cosmetics Safety and Efficacy Evaluation Center, West China Hospital, Sichuan University, Chengdu, China; ^3^Department of Dermatology, West China Hospital, Sichuan University, Chengdu, China; ^4^Department of Cardiology, Sichuan Academy of Medical Science and Sichuan Provincial People's Hospital, University of Electronic Science and Technology of China, Chengdu, China; ^5^Institute of Dermatology and Venereology, Sichuan Academy of Medical Science and Sichuan Provincial People's Hospital, University of Electronic Science and Technology of China, Chengdu, China

**Keywords:** COVID-19 pandemic, online education, MOOCs, digital divide, cosmetic, socioeconomic distribution

## Abstract

**Objectives:** During the pandemic, quarantine has led to the lockdown of many physical educational institutions. Thus, massive open online courses (MOOCs) have become a more common choice for participants. MOOCs are often flagged as supplemental methods to educational disparities caused by regional socioeconomic distribution. However, dissenters argue that MOOCs can exacerbate the digital divide. This study aimed to compare the participants' performance before and after the outbreak of COVID-19, analyze the impact of the epidemic on online education of cosmetic dermatology from the view of the regional socioeconomic distribution, and investigate whether MOOCs exacerbate the digital divide in the COVID-19 epidemic.

**Methods:** The study was conducted in participants of the MOOC course *Appreciation and Analysis of Cosmetics* from January 2018 to December 2020. Based on the platform data and official socioeconomic statistics, correlation of multivariate analysis was used to determine the factors related to the number of total participants. A panel regression model and stepwise least squares regression analysis (STEPLS) were employed to further analyze the relationship between GDP, population, number of college students and number of total participants in different years in the eastern, central and western regions of China.

**Results:** The number of total participants in 2020 surged 82.02% compared with that in 2019. Completion rates were generally stable in 2018 and 2019 before the COVID-19 pandemic and significantly decreased in 2020 after the outbreak of the pandemic. GDP was the most important socioeconomic factor that determined the total number of participants and it was positively related to the total number of participants before and after the outbreak of the pandemic. The number of college students was unrelated to the total number of participants before the epidemic, and after the outbreak of COVID-19 in 2020, the number became positively related in all regions of China.

**Conclusions:** This study shows that the epidemic pushes more people to choose MOOCs to study cosmetic dermatology, and online education could exacerbate rather than reduce disparities that are related to regional and socioeconomic status in the cosmetic field in the COVID-19 pandemic.

## Introduction

COVID-19 is both highly contagious and transmissible. As the world is in an age of widespread global trade and travel, it causes the means for a rapid spread in the disease regardless of national borders. As of June 30th, 2020, a total of 10 million confirmed cases have been detected in 212 countries (1). Governments have responded by implementing self-isolation and physical distancing measures that billions of people have adopted into their daily routines that could potentially cause negative psychological effects (2). Being in quarantine is often an unpleasant experience and leads to negative emotions, including posttraumatic stress symptoms, confusion, anxiety and anger (3–5). During this period, it is a good choice to study independently by networking to eliminate negative feelings. Currently, little evidence exists on how people are self-learning during the pandemic. Meanwhile, Chinese universities had to suspend all on-site activities (6). With the closure of all campuses, 22 online course platforms, including MOOCs (massive open online courses), Wisdom Tree and xuetangX, were organized to develop and diversify a distance-learning solution by February 2, 2020, with over 24,000 online courses and 401 national virtual simulation-based courses available for universities to choose from (7, 8). Nevertheless, online education faces the problem of an unprecedented large scale.

Here, we provided an online course about cosmetic dermatology on the MOOC platform, aiming to provide high-quality online education while fighting the epidemic. In light of the course of *Appreciation and Analysis of Cosmetic*, it probes into some professional knowledge with cosmetic-associated dermatology and enhances a person's self-cultivation for the general public. Cosmetic products are closely associated with a series of dermatoses, such as sensitive skin, contact dermatitis, atopic dermatitis and rosacea (9, 10). While we all refrain from going outdoors, people have reduced the use of make-up during the pandemic. However, products for personal care and hygiene are still used daily, including soap, shampoo and face cream. Therefore, building the capability to select suitable cosmetic products for most people is a requisite protective strategy for certain dermatoses. However, the lack of access to professional knowledge makes it impractical (11–13). Fortunately, the popularity of online education in recent years has eased this problem. MOOCs allow the public to easily access thousands of professional fields, providing platforms for the public to conduct health education (14–16). With regard to this, the drawbacks of MOOCs, such as a high dropout rate and lack of actual practice, impact the effects of online courses on the spread of knowledge (16, 17). To virtually integrate cosmetics-associated dermatological knowledge into the daily routines of the widespread susceptible population, lowering the incidence of these dermatoses and preventing their recurrence, our team carried out an online course, *Appreciation and Analysis of Cosmetics*. Between 2014 and 2020, this course was inundated with more than 400,000 applications, which highlighted the importance of healthy-looking skin for many people.

Moreover, quarantine measures in the pandemic have profoundly impacted economic development throughout the world (2). Cosmetics represent an important industry worldwide (18). The cosmetic industry encompasses several environmental, social and economic impacts that are being addressed through searching for more efficient manufacturing techniques, the reduction of waste and emissions, and the promotion of personal hygiene, allowing it to contribute to the improvement of public health while providing employment opportunities (19). Currently, the cosmetic industry is faced with the enormous task of rebuilding the battered economy. It is known that China has a massive population, weak economic foundation and uneven regional development. At present, education inequality, especially in the field of higher education, exists in regional areas for different reasons. In the first years of the 2010s, researchers heralded the possibility that MOOCs can “democratize education.” MOOCs are empirically characterized as remedies to educational disparities related to regional differences (20). “Digital divide,” which was first proposed by Attewell (21), is an economic and social inequality with regard to access to, use of, or impact of information and communication technologies. It has been reported that pandemic-induced school closures have aggravated social inequalities and that the COVID-19 pandemic crisis is widening the gap in access to formal education (22). During an epidemic outbreak, the number of registrations and completions of online education participants related to the regional economy, education resource distribution and industrial distribution have sparked attention. Given this, we took advantage of the data collected from MOOC students who participated in the *Appreciation and Analysis of Cosmetics* course, aiming to compare the participants' performance before and after the outbreak of COVID-19, analyzed the impact of the epidemic on online education of cosmetic dermatology from the view of the regional socioeconomic distribution, and investigated whether MOOCs exacerbate the digital divide in the COVID-19 epidemic.

## Materials and Methods

### Course Context

The *Appreciation and Analysis of Cosmetics* course was launched on a website named *MOOC* of Chinese Universities (https://www.icourse163.org/course/SCU-20012?tid=1206946235). The website is supported by the Ministry of Education of China and is free to the public. This 15-week course contained 15 lessons. Each lesson was divided into 3 to 5 videos, and each video was no more than 15 min.

The MOOC course comprised three main parts: manufacture of cosmetics, functions of common cosmetics and cosmetics-associated dermatology. Manufacture of cosmetics included 2 lessons: materials, formulations and processes. Functions of common cosmetics included 11 lessons: moisturizers, oil-control products, cleaning products, hair-care products, sunscreen, whitening products, anti-inflammation products, anti-aging products, perfumes, masque and make-up products. Cosmetics-associated dermatology included 4 lessons: capability building of differentiating skin types and choosing suitable products, adverse effects of cosmetics, and approaches to deal with adverse effects.

### Evaluation of Participants

Participants were predicted to allocate 1.5 h per week on the course's contents and assignments. Participants who studied all the lessons and spent more than 1.5 h per week for 15 weeks were considered to complete the course. Final scores were determined by the grades of participants' homework (30%), quizzes (20%), the final exam (40%) and activities at the course forum (10%). With a score of more than 60, participants were considered to pass the course, and those who received more than 85 were considered to perform excellently in the course.

### Data Acquisition

Data of the years of 2018, 2019 and 2020 were collected, respectively. Data of the year of 2018 and 2019 were considered to represent the situation before the outbreak of COVID-19, and those of 2020 were after the pandemic. The numbers of total participants, personal information (including age, gender, address, educational background) of the participants, completion rates, pass rates and rates of excellent performance of each year of the *Appreciation and Analysis of Cosmetics* course were obtained from the MOOC platform. Regional economic data, including gross domestic product (GDP), gross population, consumer price index (CPI) and numbers of higher institutions along with numbers of college students, were acquired from the National Bureau of Statistics of China (http://www.stats.gov.cn). We obtained the numbers and locations of cosmetic manufacturing companies that owned a production license from the National Medical Products Administration of China (https://www.nmpa.gov.cn).

### Data Analysis

Stata 15.1 software (StataCorp, College Station, TX, USA) was used for data analysis. The differences in completion rates, pass rates and rates of excellent performance between 2018 and 2019, 2018 and 2020, 2019 and 2020 were compared by the Chi-square test. Correlations between the number of participants and socioeconomic factors were analyzed using Pearson correlation test. Data were divided into eastern, central and western regions for further analysis. Panel data regressive analysis and stepwise least squares regression analysis (STEPLS) from three regions were undertaken. For the chi-square test, *p* < 0.01 was considered significantly different. The stronger the tendency was, the larger the absolute value of the correlation coefficient (R value) in the Pearson correlation test. For panel data regressive analysis and STEPLS, *p* < 0.1 was significantly different, and the correlation coefficient value represented whether there was a positive or negative correlation but did not reflect the strength of the correlation between variables.

This study employed a panel regression model to analyze the relationship between the number of participants and regional socioeconomic distribution. Panel data have both temporal and cross-sectional dimensions. This method can overcome the interference of multicollinearity in time series analysis, ensuring that regression results are reliable. In this study, a random effect regression model acted as the reference for our theoretical framework.

This model can be written as Eq:


$$\begin{array}{l} \text{Y}={\text{β}}_{0}+\beta_{1}\text{GD}{\text{P}}_{\text{it}}+\beta_{2}\text{populatio}{\text{n}}_{\text{it}}+\beta_{3}\text{studen}{\text{t}}_{\text{it}}+\varepsilon_{\text{it}} \end{array}$$

where Y represents the number of participants; β_0_ is a constant term; GDP, population and student represent GDP, population and number of college students in each region, respectively; β_1_, β_2_ and β_3_ are the coefficients of the three explanatory variables; i represents individuals, i.e., each region; and *t* stands for years (2018, 2019 and 2020).

## Results

**Figure 1:**
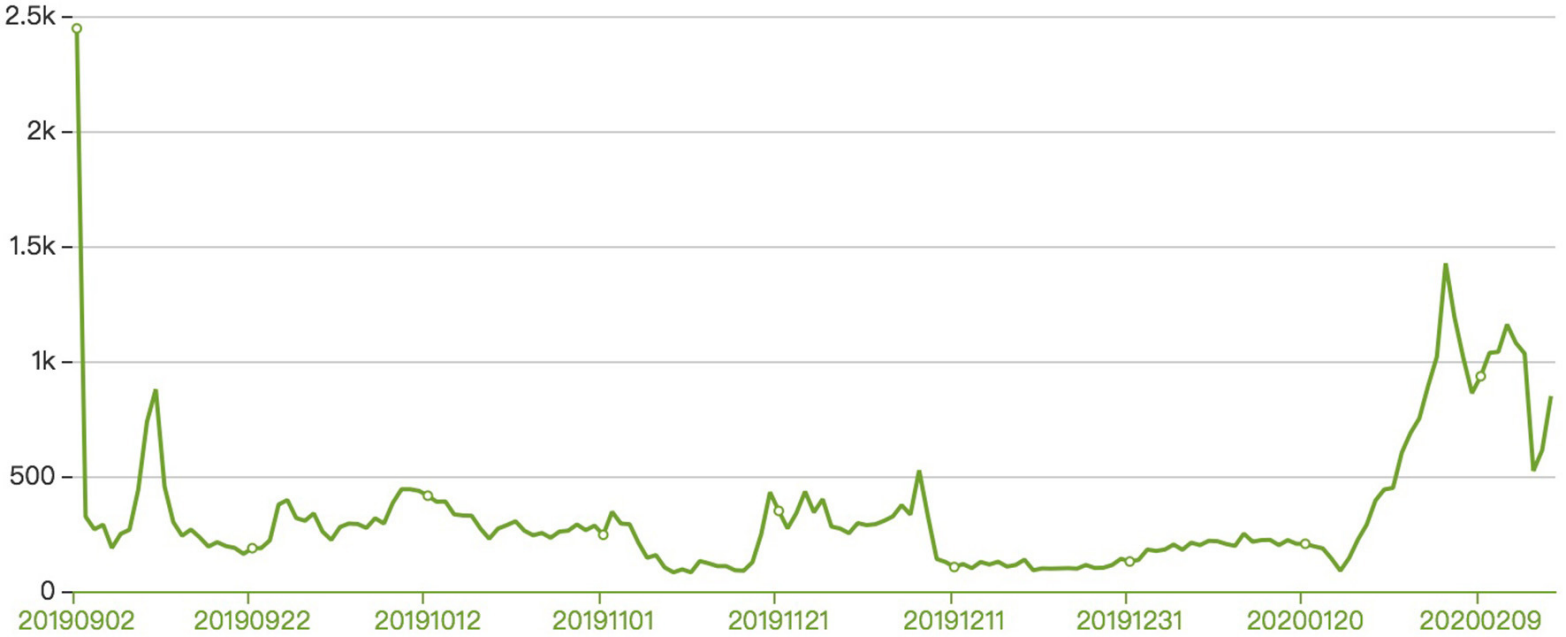
Applications from January 31 to February 14 of 2020.

A total of 120,359 individuals were enrolled. 76.92% of the registered participants were female, and 23.08% were male. 15.69% got pre-university education, 52.27% were college students, 18.73% had a bachelor's degree and 13.32% had a master's degree. The majority of students lived in Jiangsu (15.34%), Henan (9.21%), Guangdong (8.67%), Zhejiang (5.94%), Beijing (5.87%) and Shandong (5.34%). There were 34,774 participants who took part in the course in 2018, 30,348 in 2019, and 55,237 in 2020. The number of total participants in 2020 surged 82.02% compared with that in 2019. Data from the MOOC platform also showed that there were never more than 250 new registered participants between December 9th, 2019, and January 24th, 2020. However, the number increased sharply on January 25th, 1 day after Wuhan told all nonessential businesses to close. The course was inundated with more than 600 participants a day after isolation measures were taken because of the COVID-19 pandemic, and there were 14,125 new participants in total from January 31 to February 14 of 2020 (Figure 1).

Performance of participants is shown in Table 1. A total of 2,757 (7.93%) participants completed the course in 2018, 2,377 (7.83%) completed it in 2019 and 3,056 (5.53%) completed it in 2020. Chi-square analysis documented that completion rates were generally stable in 2018 and 2019 (χ^2^ = 0.190, *p* = 0.663) before the COVID-19 pandemic and significantly decreased in 2020 after the outbreak of the pandemic.

**Table 1:** Performance of course participants in different years.

	**2018**	**2019**	**2020**
Number of participants	34,774	30,348	55,237
Completion rate (number)	7.93% (,2757)	7.83% (2,377)	5.53% (3,056)
Pass rate (number)	1.65% (574)	1.25% (378)	1.25% (691)
Rate of excellent performance (number)	0.54% (188)	0.70% (212)	0.79% (463)

Participants were from 31 provinces of China. Correlations between the number of participants and socioeconomic factors of 31 provinces in 2018, 2019 and 2020 were analyzed using the Pearson correlation test. The results showed that all factors (GDP, population, CPI, number of certified cosmetic manufacturing companies, number of universities, number of college students) were positively correlated with the number of applicants (Table 2). For each year, GDP, population and number of college students were the top 3 factors mostly related to the number of participants.

**Table 2:** Correlation between the number of participants and socioeconomic factors across 31 provinces of China.

	**2018 Coeff.**	**2019** **Coeff.**	**2020 Coeff.**
GDP	0.8795	0.8400	0.8160
Population	0.7314	0.7604	0.6677
Number of college students	0.7212	0.7980	0.6777
Number of universities	0.6617	0.7254	0.5954
CPI	0.5531	0.3333	0.4043
Number of certified cosmetic manufacturing companies	0.5214	0.4829	0.4134

*Coeff., correlation coefficient (R value); the stronger the tendency was, the larger the R value*.

The STEPLS was used to estimate the effects of the top 3 socioeconomic factors (GDP, population, and number of college students) on the number of participants in different regions of China in different years (Table 3). The results revealed that GDP was positively related to the number of participants in the whole region, no matter before (2018 and 2019) or after (2020) the outbreak of the COVID-19 pandemic, which was consistent with the Pearson correlation test results. The number of participants had a sustainable positive relationship with GDP in eastern China from 2018 to 2020, but in other regions, the numbers were not always associated with GDP. Before the pandemic in 2018 and 2019, the number of college students was not related to the number of course participants. However, after the outbreak of the pandemic in 2020, the number of college students had a positive correlation with the number of participants, especially in the central and western regions.

**Table 3:** Effects of GDP, population, and number of college students on the number of participants in different regions of China in different years.

**Year**	**Socioeconomic factor**	**Whole region Coeff.**	**Eastern region Coeff.**	**Central region Coeff.**	**Western region Coeff.**
2018	GDP	0.0362***	0.0307***	N/A	0.0499***
	Population	N/A	N/A	0.204***	N/A
	Number of college students	N/A	N/A	N/A	N/A
2019	GDP	0.0313***	0.0367***	N/A	N/A
	Population	−0.288**	−0.356***	N/A	−0.564***
	Number of college students	N/A	N/A	18.40***	N/A
2020	GDP	0.109***	0.125**	N/A	N/A
	Population	−0.896**	−1.639*	N/A	N/A
	Number of college students	35.37*	74.85*	36.02***	7.041**

*Coeff., correlation coefficient*;

*** *p < 0.01*;

** *p < 0.05*;

* *p < 0.1; N/A, not available, uncorrelated*.

Table 4 presents the regression results in relation to the panel data. In general, the effects of the three main socioeconomic factors (GDP, population and number of college students) of the eastern region are consistent with those of the whole region. The correlation coefficient of the number of participants and GDP in the eastern region was significantly higher than those in the central and western regions, reflecting the effect of GDP on education inequality. The results demonstrated a negative correlation between the population of each region and the number of participants. The number of college students, population and GDP were all positively correlated with the number of participants in the eastern region. The results indicated that the quality of education and the economic level within the population exerted a positive effect on online courses in the east. The number of college students was positively correlated with the number of participants in the central and western regions, while population and GDP were negatively related.

**Table 4:** Results of the panel data regression analysis for different variables.

**Socioeconomic factor**	**Whole region Coeff.**	**Eastern region Coeff.**	**Central region Coeff.**	**Western region Coeff.**
GDP	0.0634***	0.0705***	−0.0684	−0.00215
Population	−0.529***	−0.862***	−0.297	−0.0889
Number of college students	24.01***	41.98***	56.23***	14.20**

*Coeff., correlation coefficient*;

*** *p <0.01*;

** *p <0.05*.

## Discussion

In recent years, online commerce, banking, and social networks have changed the way we shop, manage our lives, and meet and communicate with each other. Compared with the 2003 outbreak of severe acute respiratory syndrome (SARS), citizens were better equipped to cope with quarantine in an epidemic. Notably, we raise concerns regarding whether convenient network life and online learning can exacerbate rather than reduce disparities in resource distribution among regions related to socioeconomic status during the COVID-19 pandemic.

This study showed that the number of individuals enrolled in the cosmetic dermatology online course almost doubled after the outbreak of COVID-19. The COVID-19 pandemic indeed attracted more people to choose online courses as their way of learning. The COVID-19 pandemic has caused a lockdown situation impeding all educational institutions. This circumstance demanded online classes as an alternative strategy for the continuation of education. Many people who would not study on the internet turned to choose online education during the pandemic. Participants may choose MOOCs out of various reasons. Apart from the self-motivated reason, socioeconomic factors also play an important role in their decisions (20, 23, 24).

The completion rates were relatively low each year, which was consistent with data reported in the literature (23). Many factors may induce a low completion rate, such as the course length and design, the flexible learning form of MOOCs, the complicated background of participants and their ability to access the needed technology for MOOCs and so on (25). The completion rate in 2020 was significantly lower than those in 2018 and 2019. The current era of information overload, especially in the quarantine during a pandemic, can also create a multitude of distractions and obstacles for participants to complete an online course. Participants from a more diverse background after the outbreak of the COVID-19 pandemic tended to have less patience, and the ability to complete the course might also be a reason.

The unbalanced development of the regional economy is a common problem in all countries around the world, including China. With China's vast land and abundant resources, regional socioeconomic distribution has been driven by a boom in natural resources. The existing studies were based on individual socioeconomic status and educational attainment. A previous study conducted by Harvard and MIT between 2012 and 2014 reported that MOOC participants tended to live in more affluent and better-educated neighborhoods than the average U.S. resident (20). Studies have also reported that telemedicine could be helpful in the current pandemic, but organizational readiness to adopt telemedicine needs urgent attention (24, 26). Does digital divide exist despite broad accessibility of mobile tools and internet use in China? If it exists, does the COVID-19 pandemic increase it?

We evaluated regional economy (GDP, CPI), higher education distribution (number of higher institutions and college students), cosmetic industry distribution (number of certified cosmetic manufacturing companies) and population as potential confounders between the relationship with our cosmetic dermatology course participants in different years, including years before (2018 and 2019) and after the pandemic (2020). The results of the Pearson correlation test showed that all the above factors were positively correlated with the number of participants. GDP, population and the number of college students were the top 3 most related socioeconomic factors. Panel data regression analysis and STEPLS also revealed that GDP and the number of college students were positively related to the number of participants in the whole region of China.

Population is an important socioeconomic factor. Inequalities exist between different populations depending upon their geographical locations. In rural areas with a small population, large investments and diminishing profits make building broadband infrastructure an unattractive investment (27). Online education could also be affected by population. The results of the Pearson correlation test of our study showed that the correlation between the number of students and the population was strongly positive each year. However, panel data regression analysis and STEPLS revealed the opposite result. Panel data have both temporal and cross-sectional dimensions. This method can eliminate the problems caused by strong correlation and collinearity between data. Thus, we think that the positive correlation between the population and the number of students is not reliable. The population may not be a suitable factor for the analysis of this study because the population is enormous and the number of participants of the MOOC course is too small.

GDP, the most commonly reported measure of aggregate output, is the market value of all final goods and services produced and is representative of the level of general welfare (28–30). Sometimes, GDP means that the government might give priority to regional construction in allocating education, economy and infrastructure resources (28). GDP is abetted in part by resource distribution and government decisions whose impact is considered by the population density. In other words, the economic future of most regional areas will be determined by the productivity of these burgeoning populations. It is necessary to provide demographic approval for the implementation of a higher education strategy in China from demographic and economic perspectives. Previous studies have demonstrated that MOOCs and similar approaches cannot “democratize education” as a change in structure within an individual's socioeconomic status (1, 3, 10, 31). The results of our study inferred that GDP might impact the number of MOOC participants. The majority of participants of our MOOC course were from Jiangsu, a developed province of coastal areas in China, with its people relatively richer and further educated (1, 3, 32). As a province with rapid economic development, Guangdong had the third most registered participants, holding the leading position in both GDP and financial revenue (33, 34). Taking into consideration China's large surface area and the uneven distribution of regional development, we further analyzed data from different regions (eastern, central and western), which might provide us with more information on the correlation of participants and socioeconomic factors. The eastern region is resource-poor, the central and western regions are resource-rich (32). Previous literature has consistently confirmed the negative impact of natural resource dependence on public education expenditure (35–37). The eastern region is better developed and tends to spend more education expenditure than other regions. Our study showed that the number of participants had a sustainable positive relationship with GDP in eastern China from 2018 to 2020, which was consistent with the results in the whole region. However, in other regions, the numbers were not always associated with GDP. We speculated that this was because participants from the eastern region predominated.

Education is a major factor contributing to economically sustainable development due to its potential for improving cognition and skill levels and therefore enhancing worker productivity. Our study showed that there was a positive correlation between the number of MOOC participants and the number of college students, which inferred that MOOCs could exacerbate the digital divide. MOOCs have been reported to favor participants with higher education (23). Previous studies have also found that MOOC students have the advantages of higher educational credentials (38) and that most MOOC students are graduates with a bachelor's degree, while the remainder are older “continuing learners” (16, 39–41). There are even some interesting studies suggesting that parents' literacy has an influence on the completion rates of MOOCs (20). This study revealed that economic conditions and education are explanatory factors with a seemingly greater significance and impact on online learning in provinces as well as other administrative regions with higher levels of economic development. The panel regression model and stepwise least squares regression analysis (STEPLS) also showed that before the pandemic in 2018 and 2019, the number of college students was not related to the number of course participants. However, after the outbreak of the pandemic in 2020, the number of college students had a positive correlation with the number of participants, especially in the central and western regions. It has been reported that online courses play an important role in student isolation at home (42). Our results further indicated that MOOCs had become a mainstream method for college students during the pandemic. The number of college students became positively related to the number of participants during the pandemic also implying that the pandemic could increase the digital divide.

## Conclusions

In conclusion, this study investigated an online cosmetic dermatology course and compared data of course participants and socioeconomic factors before and after the COVID-19 pandemic. The results showed that the epidemic attracted more people to choose online courses. GDP was the most important socioeconomic factor that determined the total number of participants and it was positively related to the total number of participants in the whole region before and after the outbreak of the pandemic. The number of college students was unrelated to the total number of participants before the epidemic, and after the outbreak of COVID-19 in 2020, the number became positively related in all regions of China, which prompted MOOCs to become a mainstream study method for college students in the pandemic and implied that the pandemic increased the digital divide. Our study could enrich the public in regard to online education on cosmetic-associated dermatological knowledge from the view of the regional socioeconomic distribution.

## Data Availability Statement

The original contributions presented in the study are included in the article/Supplementary Material, further inquiries can be directed to the corresponding author.

## Author Contributions

MS, LX, and YC: formal analysis. MS and LX: writing—original draught preparation. LL and YM: conceptualisation and project administration. LX, LL, JT, and WH: resources and data curation. YC and YM: writing—review and editing. All authors contributed to the article and approved the submitted version.

## Funding

This work is supported by the Science Foundation of Sichuan Provincial People's Hospital (2018QN02) and Foundation of Science and Technology Department of Sichuan Province (2021YFS0201).

## Conflict of Interest

The authors declare that the research was conducted in the absence of any commercial or financial relationships that could be construed as a potential conflict of interest.

## Publisher's Note

All claims expressed in this article are solely those of the authors and do not necessarily represent those of their affiliated organizations, or those of the publisher, the editors and the reviewers. Any product that may be evaluated in this article, or claim that may be made by its manufacturer, is not guaranteed or endorsed by the publisher.
