# Supplementary material for: Digital Divide in Online Education During the COVID-19 Pandemic: A Cosmetic Course From the View of the Regional Socioeconomic Distribution
Source: Front Public Health. 2022 Jan 3;9:796210. doi: 10.3389/fpubh.2021.796210 (PMC8761946; doi:10.3389/fpubh.2021.796210)
Supplement: Supplementary file 1 [file Table_1.DOCX]

**TABLE 1S** | Fourfold table

| Year | Completion | Not completion | Summary | Completion rate |
| --- | --- | --- | --- | --- |
| 2018 | 2757 | 32017 | 34774 | 7.93% |
| 2019 | 2377 | 27971 | 30348 | 7.83% |
| Summary | 5134 | 59988 | 65122 | 7.88% |
